# Coverage of overdose prevention programs for opiate users and injectors: a cross-sectional study

**DOI:** 10.1186/1477-7517-11-33

**Published:** 2014-11-22

**Authors:** Elisabet Arribas-Ibar, Albert Sánchez-Niubò, Xavier Majó, Antònia Domingo-Salvany, Maria Teresa Brugal

**Affiliations:** Research group of drug abuse epidemiology, IMIM—Institut Hospital del Mar d’Investigacions Mèdiques, Doctor Aiguader 88, E-08003 Barcelona, Spain; Public Health Agency of Catalonia, Edifici Salvany, Roc Boronat 81-95, 08005 Barcelona, Spain; Public Health Agency of Barcelona, Pl. Lesseps 1, 08023 Barcelona, Spain

**Keywords:** Coverage, Drug injectors, Harm reduction program, Heroin overdose, Opiates users, Drug and overdose prevention program

## Abstract

**Background:**

The use of opiates, particularly heroin, remains an important cause of morbidity and mortality. Half of the deaths among heroin consumers are attributed to overdose. In response to this problem, overdose prevention programs (OPPs) were designed. The objective of our study was to assess coverage of OPPs among the target population in a specific Spanish region (Catalonia) and to identify characteristics related to attendance.

**Methods:**

A cross-sectional survey recruited individuals from outpatient treatment centers (OTCs), therapeutic communities (ThCs), and harm reduction facilities (HRFs) in Catalonia. From 513 participants, 306 opiate users and/or injectors were selected for this study. Coverage was calculated as the proportion of subjects who declared having participated in an OPP. A Poisson regression with robust variance was used to assess factors (socio-demographic aspects and psychoactive substance use patterns) associated to OPP participation, taking into account recruitment strategy.

**Results:**

Average age of the 306 subjects was 39.7 years (s.d.: 7.7); 79% were male; 79.2% lived in urban areas and 56.3% were unemployed or had never worked. Overall OPP coverage was 43.5% (95% CI: 37%–49%). Training was received mostly in HRF (60%), followed by OTC (24.4%), prison (19%), and ThC (16%). OPP sessions were attended by 41% of Spanish-born study participants and by 63.3% of foreigners; 92.2% of the participants lived in urban areas. The Poisson regression analysis adjusted by age, sex, and type of recruitment center showed that OPP participation rates were higher for individuals with foreign nationality (PR = 1.3; 95% CI: 1.04–1.72), for those living in municipalities with more than 100,000 inhabitants (PR = 2.0; 95% CI: 1.37–2.81) or the Barcelona conurbation (PR = 2.5; 95% CI: 1.68–3.77), and for those having ever been in prison (PR = 1.6; 95% CI: 1.41–1.81) and had first consumption when they were less than 12 years old (PR = 1.2; 95% CI: 1.06–1.45).

**Conclusion:**

Coverage as a whole can be considered high. However, in Catalonia, new strategies ought to be developed in order to attract opiate users and injectors not currently participating, by expanding OPP offer to services and regions where coverage is poor.

## Abstract (es)

**Antecedentes:**

El uso de opiáceos, sobre todo heroína, sigue siendo una causa importante de morbilidad y mortalidad. La mitad de las muertes entre consumidores de heroína se atribuyen a sobredosis. Como respuesta a este problema, se desarrollaron los Programas de Prevención de Sobredosis (PPS). El objetivo fue evaluar la cobertura de PPS e identificar las características relacionadas con la asistencia.

**Métodos:**

Estudio transversal de consumidores reclutados en Centros de Tratamiento (CT), Comunidades Terapéuticas (CoT) y Centros de Reducción de Daños (CRD) en Cataluña (España). A partir de 513 participantes, se seleccionaron 306 consumidores de opiáceos y/o inyectores. La cobertura fue calculada como la proporción de sujetos que declararon haber participado en un PPS. Se realizó una regresión de Poisson con varianza robusta para evaluar los factores (aspectos socio-demográficos y patrones de consumo de drogas), asociados a la participación en PPS teniendo en cuenta la estrategia de reclutamiento.

**Resultados:**

La edad media fue de 39.7 años (de: 7.7); 79% eran hombres; 79.2% vivían en zonas urbanas y 56.3% eran desempleados o que nunca habían trabajado. En general, la cobertura de PPS fue del 43.5% (IC 95%: 37%-49%). La mayoría de los programas se realizaron en CRD (60%), seguido por CT (24.4%), cárcel (19%) y CoT (16%). El 41% de los participantes nacidos en España participaron en un PPS mientras que lo hicieron un 63.3% de los extranjeros; el 92.2% de los participantes vivían en zonas urbanas. Se realizó regresión de Poisson para el análisis, ajustado por edad, sexo y centro de reclutamiento. Se obtuvieron mayores tasas de participación en PPS en personas nacidas en el extranjero (RP = 1.3; 95% IC: 1.04-1.72), residentes en municipios con más de 100.000 (RP = 2.0; 95% IC: 1.37-2.81) y Barcelona conurbación (RP = 2.5; 95% IC: 1.68-3.77), en aquellos que habían estado alguna vez en la cárcel (RP = 1.6; 95% IC: 1.41-1.81) y en los que hicieron el primer consumo con menos de 12 años (RP = 1.2; 95% IC: 1.06-1.45).

**Conclusión:**

La cobertura puede ser considerada alta, sin embargo en Cataluña nuevas estrategias deben ser desarrolladas con el fin de atraer consumidores de opiáceos y/o inyectores que no participan, mediante la ampliación de la oferta de PPS en los centros y las regiones donde la cobertura es deficiente.

## Background

Currently, substance abuse remains a huge public health problem worldwide, despite different measures having been undertaken in terms of prevention and treatment for legal and illegal drugs [1]. It is estimated that globally, over 27 million people are affected by problematic consumption of some kind of substance (0.6%). In numerous developed countries, illicit drug use rates are alarming, opiates being one of main substances involved [1–4]. The European Commission and the EU drugs agency (EMCDDA) have estimated that in 2010, there were over 1.3 million active opiate users in Europe [5] and the average prevalence of problematic use is estimated to be between 3.9 and 4.4 individuals per 1,000 persons aged 15–64 years [2]. Furthermore, heroin has been classified as the second most harmful psychoactive drug, taking into account harm to self and others [6], and remains an important cause of morbidity and mortality [2]. Different studies have estimated that around half of the deaths of heroin consumers may be attributed to overdose [7], even though a cohort study in Barcelona found that 34.7% were due to opiate overdose, probably in relation to the high AIDS-related mortality there [8]. However, it has been estimated that only around 2%–3% of heroin overdoses are fatal [9]. On the other hand, studies have shown that the annual prevalence of non-fatal overdose episodes is 9%–32% [9, 10]. Even when non-fatal, they can still have serious health consequences [10, 11].

Circumstances and consequences of consumption are almost always involved in heroin-related deaths [12]. The risk of heroin overdose, whether fatal or non-fatal, is related to factors such as parenteral route of administration [13] and polydrug use [2, 7], especially with central nervous system depressors (e.g. alcohol, benzodiazepines) [10, 14, 15]. Using heroin when alone and using heroin after a period of abstinence have been established as risk factors for fatal opiate overdose [12, 16].

To reduce the health impact that heroin use entails, different strategies have been developed. In several countries, treatment and prevention programs were set up to diminish heroin-associated harm and mortality [7, 14]. Among other therapeutic approaches, substitution treatment programs have been highly valuable, with methadone substitution being one of the most widely employed. Methadone maintenance programs have been shown to reduce incidence of both fatal and non-fatal heroin overdoses [8, 17]. Despite the effectiveness of these programs, a considerable number of heroin addicts, whether or not enrolled in them, continue heroin use and thus remain at an increased risk of suffering from health problems, including overdose. Harm reduction programs were created to reduce fatal and non-fatal consequences of heroin use. They include needle exchange programs, safe injection sites, naloxone distribution, and overdose prevention programs (OPPs) [14].

Accidental overdoses usually occur in the company of peers whose immediate help may be critical to assist the overdose victim. Furthermore, as death from overdose usually occurs within 1–3 h after heroin injection [14, 18], there is time to act in order to avoid the opiate poisoning being fatal. This fact led to the development of overdose prevention programs (OPPs), special courses designed [14, 18] to teach opiate users how to avoid suffering an overdose and train them in treating those of their peers [19]. The OPP consist of explaining the risks and consequences of opiate use, as well as teaching participants to recognize signs of overdose, how to deal with an overdose step by step, and instruction in cardiopulmonary resuscitation technique and naloxone administration [14, 19–21]. Naloxone as an opiate antagonist is very useful to reverse an opiate overdose and as several feasibility studies have demonstrated its effectiveness [22], training in its administration has been incorporated into OPP. Prior studies have shown that drug users trained in OPP are very effective in recognizing overdose signs and in reducing overdose deaths [23].

In Catalonia (Spain), opiate use by injection continues to be prevalent [24]. The region has been identified as having a high mortality rate for opiate overdose [25]. Many treatment and prevention programs started first in Barcelona and subsequently expanded, mainly to other urban areas. Implementation of OPP appears to have followed a similar pattern until their systematic application started, first in centers where harm reduction programs are offered (2009), followed by therapeutic communities (2010) and outpatient treatment centers (2011) [XM, personal communication]. These programs last around 1 h and participants receive an economic reward. Coverage of these programs needs to be evaluated in order to guarantee their adequacy [23]. In this sense, the main aim of the present study was to assess coverage of OPPs among the target population and to identify characteristics related to attendance.

## Methods

### Study design and sample

The sample for this cross-sectional study was recruited in treatment and prevention centers in Catalonia (Spain) between April and June 2012. A total of 48 centers were selected: 26 outpatient treatment centers (OTCs), 12 therapeutic communities (ThCs), and 10 harm reduction facilities (HRFs). Sampling was planned to cover the whole territory, each center was required to recruit a number of participants determined based on their activity, over-sampling the smallest selected centers, especially HRF. In OTC, this was done only for those having more than 45 annual patients; and in each OTC, a convenience sample was selected, taking into account whether first treatment and time in treatment. Approval for the study was obtained from the IMIM (Hospital del Mar Medical Research Institute) ethics committee, and ethical procedures were followed for data collection. Participants were informed of the purpose of the study, and HRF clients were rewarded with 10 €.

From a sample of 513 drug users (*N* approached = 564; participation rate: 91%), the target population was selected for the present study, consisting of 312 opiate (i.e. heroin, methadone) users and/or injectors. Participants answered a 78-item questionnaire involving single or multiple-choice answers and different levels of complexity. It was clearly structured and asked for information about socio-demographic aspects, drug patterns, illegal drug market activities, violence, health status, and evaluation of prevention programs. Most questionnaires were administered by a previously trained interviewer; a minority of participants completed the questionnaires themselves. Only four participants did not answer questions about participation in OPPs and two didn’t know about participation (1.9% of the target population). Therefore the final sample consisted on 306 opiate users and/or injectors.

The coverage of OPPs was calculated for the total sample, by recruitment center, region, and municipalities, as the proportion of subjects who declared having participated in an OPP. Variables of interest for OPP participation analysis were recruitment center, socio-demographic aspects (sex, age, country, municipality, level of education, employment status, and sentenced to prison), and psychoactive substance use patterns (age at first drug use, first drug used, parenteral administration, heroin and cocaine use, alcohol risk consumption, and binge drinking). Alcohol risk consumption was assessed through the AUDIT, short version [26]. Other variables of interest were those related to OPPs (type of training center and when they had attended OPP).

### Statistical analyses

Poisson regression models with robust variance were used to analyze association of OPP attendance with the other variables mentioned above via prevalence ratios (PR), adjusted by sex and age [27]. In addition, correlated observations according to the type of recruitment center (HRF, OTC, or ThC) were taken into account through the generalized estimating equation procedure. Analyses consisted in two steps. Firstly, we used distinct Poisson regressions (bivariate) for each variable, taking non-attendance in OPP as the reference category. Later, those variables with *p*-value <0.2 were included in a multiple Poisson regression. Final model was fitted using a backward procedure. Statistical significance was set at *p*-value <0.05. All analyses were performed using PASW Statistics version 18.

## Results

Among the 306 participants, 68.3% were opiate users and injectors, 28.4% were only opiate users, and 3.3% only injectors. The majority of participants were male (79.2%). The average age was 39.7 years (s.d.: 7.7) (range: 20–71). Only 24% had high school or university degree. Over half of the participants had never worked or were unemployed (56.3%), 26.2% received a pension or had some permanent disability, and 12% were currently working; 57.3% of participants had a prison history. Regarding drug use patterns, alcohol risk consumption was observed in 45.4% of study participants; 18% started illegal drug use when they were under 13 years old; and in 76.3%, the first illegal drug used was cannabis.

One hundred thirty-three study participants had participated in some OPPs (43.5%; 95% CI: 37%–49%). No significant differences were found in the distribution of OPP participation by either sex (men 44.6% vs women 39%) or age (mean age of OPP participants: 39.4 years s.d.: 7.1); 66.7% of participants recruited in HRF had attended an OPP, while only around one-third of individuals recruited in OTC and ThC had done so (Table 1). OPP coverage by geographical area shows that people living in Barcelona city or Barcelona metropolitan conurbation (BMC) had the highest participation (52.3%) (Table 1). Related to time of attendance, 63.4% had attended within the last 2 years, 19.1% between 2–5 years ago, and 17.6% more than 5 years ago. Among 131 OPP participants reporting where they had received training, 60% did so in HRF, 24.4% in OTC, 19% in prisons, and 16% in ThC (note that they could have attended an OPP several times).

**Table 1:** **Overdose prevention program (OPP) coverage by recruitment center and geographical area**

	Attendance to OPP			
	***N***	Yes		
*Recruitment center*	*306*	*n*	*%*	*95% CI*
Outpatient treatment center	156	50	32.1	(27%–37%)
Therapeutic communities	54	19	35.2	(30%–41%)
Harm reduction facilities	96	64	66.7	(61%–72%)
*Municipality*	*305*			
Barcelona	130	66	50.8	(45%–56%)
BMC	49	29	59.2	(54%–65%)
More than 100,000 inhabitants^a^	63	27	42.9	(37%–49%)
10,001 to 100,000 inhabitants^a^	37	9	24.3	(19%–29%)
Up to 10,000 inhabitants^a^	26	2	7.7	(5%–11%)
*Provinces*	*300*			
Barcelona and BMC	176	92	52.3	(47%–58%)
Barcelona^a^	56	19	33.9	(29%–39%)
Tarragona	28	4	14.3	(10%–18%)
Lleida	20	10	50	(44%–56%)
Girona	20	5	25	(20%–30%)

*BMC* Barcelona metropolitan conurbation.

^a^Without Barcelona or BMC.

Regarding the associations in the bivariate analyses, between OPP participation and other individual variables (Table 2), socio-demographic characteristics found to be significantly associated to OPP attendance were: being a foreigner (PR = 1.5; 95% CI: 1.12–2.03); municipality, as residents from Barcelona and towns of more than 100,000 habitants had a higher participation; employment status as individuals with permanent disability or receiving a pension were more likely to have attended an OPP and also prison as persons who had ever been sentenced to prison declared a higher participation. Regarding drug use-related variables, we found that participants aged 12 years or under when they first used drugs were more likely to have enrolled in OPP (PR = 1.4; 95% CI: 1.19–1.75).

**Table 2:** **Socio-demographic and psychoactive substance use patterns associated to participation in overdose prevention programs (OPPs)**

	Attendance to OPP			Bivariate poisson		Multiple poisson	
				Regression ^a^		Regression ^a^	
	***N***	Yes		PR	95% CI	PR	95% CI
		***n***	%				
*Socio-demographic characteristics*							
*Country of origin*	*306*						
Spain	273	112	41	1		1	
Other countries^b^	33	21	63.6	1.5	(1.12–2.03)^*^	1.3	(1.04–1.72)^*^
*Municipality*	*305*						
Less than 100,000 inhabitants; not BMC	63	11	17.5	1		1	
More than 100,000 inhabitants; not BMC	52	20	38.5	2.2	(1.49–3.23)^*^	2.0	(1.37–2.81)^*^
Barcelona and BMC	190	102	53.7	3.1	(1.98–4.84)^*^	2.5	(1.68–3.77)^*^
*Level of education*	*306*						
High school/university degree	73	26	35.6	1			
Secondary education	121	55	45.5	1.3	(0.87–1.81)		
Primary/elementary	112	52	46.4	1.3	(1.01–1.89)^*^		
*Employment status*	*302*						
Working	36	7	19.4	1			
Never worked/unemployed	170	77	45.3	2.4	(0.95–6.02)		
Permanent disability/pensioner	79	40	50.6	2.9	(1.23–6.60)^*^		
Student/sporadic work/working at home	17	8	47.1	2.6	(0.67–10.10)		
*Ever sentenced to prison*	*304*						
No	131	36	27.5	1		1	
Yes	173	95	54.9	2.0	(1.78–2.30)^*^	1.6	(1.41–1.81)^*^
*Psychoactive substance use patterns*							
*Age at first drug use*	*305*						
≥13 years	249	100	40.2	1		1	
≤12 years	56	33	58.9	1.4	(1.19–1.75)^*^	1.2	(1.06–1.45)^*^
*First drug used*	*304*						
Cannabis (marijuana, hashish)	232	101	43.5	1			
Cocaine, heroin, her-coca, crack	48	23	47.9	1.1	(0.88–1.44)		
Other drugs^c^	24	8	33.3	0.8	(0.40–1.43)		
*Parenteral administration*	*306*						
No	87	31	35.6	1			
Yes	219	102	46.6	1.3	(1.03–1.70)^*^		
*Heroin use*	*306*						
No	16	7	43.8	1			
Yes	290	126	43.4	1.0	(0.70–1.47)		
*Alcohol risk consumption* ^*d*^	*306*						
No	167	73	43.7	1			
Yes	139	60	43.2	0.9	(0.75–1.24)		

*CI* confidence interval, *PR* prevalence ratio, *BMC* Barcelona metropolitan conurbation.

*p <0.05.

^a^Poisson regression with robust variance adjusted by age and sex, using generalized estimated equations to control correlation within recruitment center.

^b^Other countries: rest of Europe, America, Asia, and North Africa.

^c^Other drugs: include tranquilizers, mushrooms, LSD, and ketamine.

^d^According to AUDIT [26].

The multiple Poisson regression analysis showed that being a foreigner was associated to a greater chance of participating (PR = 1.3; 95% CI: 1.04–1.72), as was residing in a town with more than 100,000 habitants (PR = 2.0; 95% CI: 1.37–2.81) or the Barcelona conurbation (PR = 2.5; 95% CI: 1.68–3.77); also, individuals ever sentenced to prison were more likely to have participated in OPP (PR = 1.6; 95% CI: 1.41–1.81), as were subjects whose first drug use was when they were 12 years old or under (PR = 1.2; 95% CI: 1.06–1.45).

## Discussion

Participation in overdose prevention programs by the target study population was high, 43.5%. Coverage was higher in Barcelona city and its conurbation. Characteristics of participants associated to higher participation in OPP in Catalonia were: residing in metropolitan areas, being a foreigner, having been in prison, and starting consumption at age 12 years or under.

In this study, an attempt was made to achieve maximum representativeness of the target population, selecting participants from all over Catalonia, but as recruitment was only done in health centers, results can at most be representative of drug users in healthcare settings. We need to assume that the interviewed users are similar to their peers. Injectors not reporting opiate use were also considered target population because intravenous illegal drug use in Catalonia is strongly associated with heroin use [24]. Nevertheless, the resulting sample might be too small to assess coverage precisely or to ascertain the association of some variables. Another limitation would be related to information and recall biases as reporting could be influenced by desirability responses. However, some drug user studies have shown that cross-sectional results are valid despite being self-reported [28].

To our knowledge, no other studies have investigated the coverage of overdose prevention programs in a given geographical area. Coverage of other harm reduction strategies in Spain was ascertained by Barrio et al., concluding that implementation of such strategies arrived late [29]. OPP could not be examined at that point but as, in Catalonia, their systematic implementation did not begin until 2009, we can affirm that they also arrived late. However, in the present study, estimated mean coverage was above 40%, considered high by WHO, UNODC, and UNAIDS Technical Guide for clients of harm reduction programs receiving information, education, and communication [30]. Nevertheless, in this study, we found that some non-systematic provision of such programs took place in our region before 2009. In this respect, we would like to emphasize the importance of systematic implementation of these programs as almost two-thirds (63%) of the study participants had done so in the years when it was systematic. In 2009, programs were regulated in terms of education materials (e.g. videos) and professional training; however, data from the present study reflects the progressive incorporation of HRF, ThC, and OTC, participation being high only in HRF.

OPP coverage by regions and municipalities was unequal. It was much higher in large cities, not only Barcelona city and conurbation; as other cities with more than 100,000 inhabitants had a coverage of nearly 43% which could be considered high. In Catalonia, HRF are mainly located in large metropolitan areas. As in other places [31], the higher concentration of drug users in large urban areas prompted the setting up of these preventive interventions there. Starting OPP in HRF may also be considered coherent with the perceived need of OPP there, as such facilities take care of users currently using the drug. Nevertheless, such interventions should also be offered in OTC, ThC, and prisons in order to prevent, and provide the skills to assist, unexpected overdoses when resuming after a period of abstinence [32, 33]. The fact that more of our subjects recruited in HRF declared having participated in OPP and that HRF were the facilities where higher OPP attendance was reported by participants is a consistent finding (the type of center where subjects had received the OPP was correlated with recruitment center, data not shown).

OPP is considered an important support tool for overdose prevention policies which aim at reducing or avoiding deaths or health consequences. Previous studies have looked at the effectiveness of OPP, evaluating knowledge, skills learned, and drug prescription among attendants [22, 34, 35]. Different studies indicate that the vast majority of participants are ready to cope with an overdose after having participated in an OPP [36, 23]. One study found that 96% of drug users who were trained to identify overdose risk symptoms and administer naloxone treatment reported positive outcomes, such as avoiding death, coma, and brain damage [37]. Naloxone training is considered a very effective preventive measure within OPPs [23, 19]. Other studies have found that over half of the participants acquired knowledge and skills sufficient to perform rescue breathing in an overdose situation [37, 38]. The satisfaction of participants in OPP has been also assessed in some studies, finding them enthusiastic about these training programs [39] and that they feel grateful for and comfortable using the skills and tools acquired [18]. Unfortunately, in our region, there has been no evaluation of how many overdoses may have been prevented after OPP implementation.

In Catalonia, between 2009 and 2011, a total of 2,681 drug users participated in OPPs. It is interesting to note that in this study, people born abroad, with prison antecedents and those who initiated consumption early (aged under 13), reported more participation in OPP. Nearly half of the OPP participants in this study were recruited in HRF, and although recruitment center was controlled for in the regression analysis, participants’ characteristics are more similar to clients from HRF. Taking into account that HRF started OPP earlier, they are located in large cities, and most of them are open all day; such results were not unexpected. Some of the services offered in these centers, such as the so-called ‘calor-café,’ tend to attract drug users with social problems or other hardship, by allowing them to remain on the premises for a while, thus favoring their enrollment in preventive programs. This population may include immigrants and people who have been in prison. HRFs may be the first point of contact with healthcare centers and treatment for people born abroad [40, 41]. They may also be relevant for released prison inmates who learnt about the importance of harm reduction strategies while in prison [16, 33, 42]. As a whole, according to these results, so far OPPs have mainly focused on the needs of the most socially excluded, attracted by HRF, than on those of individuals having more theoretical risk (for example, drug users in OTC).

## Conclusions

Our study allowed assessing OPP coverage in Catalonia, showing that although as a whole the coverage can be considered high, less densely populated areas have a medium-poor coverage. Characteristics of OPP attenders paralleled those of HRF users, as subjects recruited there were more prone to have participated. New strategies would need to be developed to attract the target population by offering more OPP in services and regions where participation is poor and assessing why Spaniards have lower involvement in these programs.

## Abbreviations

BMC: Barcelona metropolitan conurbation
CI: Confidence interval
HRF: Harm reduction facilities
OPP: Overdose prevention programs
OTC: Outpatient treatment centers
PR: Prevalence ratio
ThC: Therapeutic communities
UNAIDS: United Nations and AIDS
UNODC: United Nations Office on Drugs and Crime
WHO: World Health Organization.
